# Safety and effectiveness of rituximab biosimilar (Rituximab-GP2013) versus originator in rheumatoid arthritis: real-world evidence from a single center

**DOI:** 10.1016/j.clinsp.2026.101040

**Published:** 2026-07-28

**Authors:** Barbara Bayeh, Fanny Reyes-Neira, Karina Rossi Bonfiglioli, Nadia Emi Aikawa, Ana Paula Luppino Assad, Renata Miossi, Fernando Henrique Carlos de Souza, Carlos Emilio Insfran, Henrique Ayres Mayrink Giardini, Emily Figueiredo Neves Yuki, Andre Franco, Diogo Souza Domiciano, Julio Cesar Bertacini de Moraes, Eloisa Bonfa, Carla Gonçalves Schahin Saad, Ana Cristina de Medeiros-Ribeiro, Andrea Yukie Shimabuco

**Affiliations:** Rheumatology Division, Hospital das Clínicas, Faculdade de Medicina, Universidade de São Paulo (HCFMUSP), São Paulo, SP, Brazil

**Keywords:** Rituximab, Biosimilar pharmaceuticals, Drug originator, Rheumatoid arthritis

## Abstract

**Objectives:**

To compare the effectiveness, safety, and retention of rituximab biosimilar (RTX-GP2013) and RTX-originator in RTX-naive Rheumatoid Arthritis (RA) patients in a real-world setting.

**Methods:**

This single-center, longitudinal, observational study included 107 RA patients who initiated RTX therapy between January/2010 and September/2023 (40 RTX-GP2013 and 67 RTX-originator). A convenience sample of consecutive eligible RTX-naïve patients was analyzed. Disease activity, treatment retention, and infusion-related reactions were assessed over six months (6M). Multivariate logistic regression identified predictors of low disease activity (CDAI ≤ 10) at 6M.

**Results:**

RTX-GP2013 patients were older (58.3 [53.1–66.4] vs. 54.9 [45.6–60.8], p = 0.029), had longer disease duration (18.4 ± 9.5 vs. 14.7 ± 8.9, p = 0.045), and a greater number of prior biologic therapies (3 [1–5] vs. 1 [0–2], p < 0.001). At 6M, frequency of low disease activity (30.0% vs. 47.8%, p = 0.103), retention rates for a second RTX cycle (77.5% vs. 85.1%, p = 0.433) and mild/moderate infusion-related reactions (17.5% vs. 9.0%, p = 0.231), were comparable between RTX-GP2013 and RTX-originator groups, respectively. Multivariate analysis identified lower baseline disease activity (per 1-point increase in CDAI: OR = 0.93, 95% CI 0.88–0.97, p = 0.003) and fewer prior advanced therapies (OR = 0.56, 95% CI 0.38–0.82, p = 0.003) as predictors of low disease activity at 6M, whereas RTX-GP2013 was not associated with poorer clinical response (OR = 1.85, 95% CI 0.63–5.45, p = 0.265).

**Conclusion:**

In this real-world study, RTX-GP2013 and RTX-originator demonstrated comparable effectiveness, safety, and treatment retention in RTX-naive RA patients. Baseline disease activity and fewer prior therapies were predictors of clinical response. These findings reinforce the role of biosimilars in expanding access to effective therapies while maintaining clinical outcomes.

## Highlights

• RTX-GP2013 showed effectiveness comparable to RTX-originator in RA.
• Safety and infusion reaction rates were similar between treatments.
• RTX retention after the first cycle was similar between RTX-GP2013 and RTX-originator.
• Lower baseline disease activity and fewer prior therapies predicted better response.
• Biosimilars may expand access to effective biologic therapy in RA.

## Introduction

Rheumatoid Arthritis (RA) is a chronic autoimmune disease that primarily affects peripheral joints, but it may involve extra-articular manifestations such as cutaneous, pulmonary, ophthalmologic, and neurologic involvement. The immune system targets the synovial membranes lining the joints, resulting in pain, swelling, and stiffness, and eventually leading to joint erosion and deformity.^1^^,^^2^ Early treatment initiation is crucial to prevent long-term joint damage. Early therapy, within the first months of symptom onset, can slow disease progression and significantly increase the likelihood of achieving remission.^1^^,^^2^

Rituximab (RTX), a monoclonal antibody targeting CD20 on B-lymphocytes, is a key biologic therapy for patients with RA who are unresponsive to conventional Disease-Modifying Antirheumatic Drugs (cDMARDs). By depleting B-cells, RTX reduces inflammation and disease activity, leading to improved clinical outcomes, prevention of structural joint damage, and enhanced quality of life. Its role remains particularly relevant in the management of severe or treatment-resistant RA, given the disease’s complex pathophysiology and heterogeneous therapeutic response.^3^, ^4^, ^5^ However, access to RTX may be limited by restrictive treatment guidelines, administrative barriers, and financial constraints, such as inadequate public funding or insurance limitations.^6^^,^^7^ In this context, the introduction of biosimilars holds promise for mitigating these obstacles. Biosimilars can facilitate earlier treatment initiation, broaden access, and improve patient outcomes.^6^, ^7^, ^8^ Robust evidence from controlled trials and systematic reviews supports the comparable efficacy and safety of RTX biosimilars,^9^, ^10^, ^11^, ^12^, ^13^, ^14^, ^15^, ^16^, ^17^, ^18^, ^19^, ^20^, ^21^, ^22^, ^23^, ^24^ including patients switched from the originator to the biosimilar.^25^, ^26^, ^27^

Despite the growing body of evidence, biosimilars still face a degree of resistance from both prescribers and patients. Although this resistance has been gradually decreasing, it can still be observed, for instance, through the so-called nocebo effect.^6^^,^^28^^,^^29^ In this context, real-world studies play a critical role in consolidating the use of biosimilars by capturing more heterogeneous clinical scenarios and the practical challenges of routine care. These studies complement randomized controlled trials and contribute to increasing healthcare professionals' confidence in prescribing biosimilars. Furthermore, they provide relevant data on long-term effectiveness, safety, and treatment retention, which can be shared with patients to support more informed and confident therapeutic decisions.^30^

Real-world data on RTX biosimilars remain limited, most available studies focus on evaluating the switch from the originator to the biosimilar and on treatment retention.^31^, ^32^, ^33^, ^34^, ^35^, ^36^, ^37^ Among those specifically assessing RTX-GP2013 (Sandoz),^31^^,^^36^^,^^37^ some include mixed populations not restricted to RA.^36^^,^^37^ These studies have shown favorable clinical outcomes with the biosimilar in both RTX-naïve patients and those who switched from the originator. However, retention and response rates appear to differ between these groups, often being higher among switchers,^31^^,^^35^^,^^36^ likely because these patients had already responded to the drug’s mechanism of action. Nevertheless, to date, no study has directly compared the clinical response or retention rates between RTX-naïve patients treated with either the RTX-GP2013 or RTX-originator in patients with RA.

To address this gap, the present real-world study aims to compare clinical response, treatment retention, and safety between the RTX-GP2013 and RTX-originator in RTX-naïve patients with RA.

## Material and methods

### Study design

This single-center, longitudinal, observational study was conducted in a cohort of patients with RA, followed between January 2010 and September 2023 at the Rheumatoid Arthritis Outpatient Clinics and the Immunobiological Drugs Infusion Center (CEDMAC – Centro de Dispensação de Medicação de Alto Custo) of the Hospital das Clínicas, Faculdade de Medicina da Universidade de São Paulo. The cohort is based on a prospective registry supported by a standardized protocol, which ensures regular clinical and laboratory evaluations every three months or as clinically indicated at this tertiary referral center for biologic therapies. The study was approved by the institutional ethics committee (CAPPesq 1298/06), and informed consent was obtained from all participants before initiating biological therapy. The reporting of this study followed the recommendations of the Strobe Statement.

A convenience sample was used, including all consecutive rituximab-naïve patients who fulfilled the inclusion criteria during the study period. Eligible patients were adults over 18-years old with RA, meeting the 2010 ACR/EULAR classification criteria,^38^ without prior use of rituximab, who received RTX for articular involvement and had at least 6-months of follow-up after the first dose of RTX infusion. Six patients were excluded from the analysis due to loss to follow-up before the minimum period, including three deaths and three treatment discontinuations.

### Treatment protocol

At our institution, biologic therapies are publicly supplied through the federal healthcare system. Until November 2020, the RTX-originator was provided. From December 2020 onward, the biosimilar RTX-GP2013 became the only RTX available and was used regularly in clinical practice. All patients received a total dose of 2g of RTX per cycle, administered as two separate infusions of 1g each, spaced two weeks apart. Standard premedication was provided prior to each infusion and included acetaminophen, intravenous methylprednisolone, and diphenhydramine, in accordance with institutional protocols to minimize infusion-related reactions.

### Data collection

Data on patients’ demographics, duration of rheumatic disease, concomitant use of glucocorticoids and DMARDs, extra-articular manifestations, and occurrence of infusion-related reactions (from the beginning up to 24-hours after the infusion), were collected. Data on RA activity parameters were collected at baseline and at 6 months (6M) after the RTX cycle. The parameters assessed included: C-Reactive Protein (CRP, mg/L), erythrocyte sedimentation rate (ESR, 1^st^ hour mm), DAS28-CRP (Disease Activity Score-28 for Rheumatoid Arthritis with CRP), DAS28-ESR (Disease Activity Score-28 for Rheumatoid Arthritis with ESR), CDAI (Clinical Disease Activity Index), and SDAI (Simple Disease Activity Index). The frequency of patients with remission/low disease activity at 6M was defined according to DAS28-CRP (< 3.2), CDAI (≤ 10), and SDAI (≤ 11).^39^ In this study, response parameters were assessed six months following a single rituximab treatment cycle.

### Statistical analysis

Categorical variables are presented as numbers (%) and compared using the chi-square test or Fisher's exact test. Continuous data are presented as means (standard deviation) or as medians (interquartile ranges) and compared using Student t-test or Mann-Whitney test, as appropriate. To identify possible predictor factors associated with low disease activity at 6M after the first RTX cycle, the variables with p < 0.10 as a result of the univariate analysis were included in the multivariable analysis. Data are presented as Odds Ratio (ORs), with 95% Confidence Interval (95% CI) and p-value. Statistical significance was considered when p < 0.05. Statistical analyses were performed using SigmaStat version 3.1 (2005) and GraphPad Prisma Software.

Because this was an observational real-world study based on a clinical registry, no formal sample size calculation was performed, and all consecutive eligible patients during the study period were included. Therefore, the analysis to assess the clinical comparability between the RTX-biosimilar and the originator should be interpreted from an exploratory perspective, and the sample size may limit the ability to detect statistically significant differences between groups.

## Results

### Baseline characteristics

A total of 107 patients were included in the evaluation of clinical response to the first cycle of RTX, comparing RTX-GP2013 (n = 40) versus RTX-originator (n = 67). Patients receiving the biosimilar were older (p = 0.029), had a longer disease duration (p = 0.045), with a similar rate of comorbidities (including smoking, obesity, and diabetes, p > 0.05) (Table 1).

**Table 1:** Demographic and clinical data of RA patients at baseline.

	RTX-GP2013	RTX-originator	
	n = 40	n = 67	p
**Age (years)**	58.3 (53.1‒66.4)	54.9 (45.6‒60.8)	**0.029**
**Disease duration (years)**	18.4 (± 9.5)	14.7 (± 8.9)	**0.045**
**Rheumatoid Factor+**	39/40 (97.5%)	64/67 (95.5%)	0.999
**Anti-CCP +**	32/34 (94.1%)	40/49 (81.6%)	0.186
**Extra-articular manifestations**	8 (20.0%)	29 (43.3%)	**0.020**
**Number of previous sDMARDs**	3 (2‒3)	3 (2‒4)	0.501
**Number of previous bDMARDs**	3 (1‒5)	1 (0‒2)	**<0.001**
**Smoking**	8 (20.0%)	10 (14.9%)	0.595
**Obesity**	5 (12.5%)	15 (20.9%)	0.305
**Diabetes**	11 (27.5%)	11 (16.4%)	0.217
**Concomitant use of:**			
Methotrexate	16 (40.0%)	24 (35.8%)	0.684
Leflunomide	13 (32.5%)	19 (28.4%)	0.999
Sulfasalazine	5 (12.5%)	6(9.0%)	0.743
Azathioprine	0	3 (4.5%)	0.291
Mycophenolate mofetil	1 (2.5%)	3 (4.5%)	0.999
Cyclosporine	1 (2.5%)	6 (9.0%)	0.252
Prednisone dose (mg/day)	6.75 (5‒12.5)	10 (5‒15)	0.152
**DAS28-ESR**	4.5 (± 1.4)	4.9 (± 1.3)	0.163
**DAS28-CRP**	4.2 (± 1.1)	4.6 (± 1.2)	0.083
**CDAI**	19.1 (± 9.5)	23.6 (± 12.1)	**0.047**
**SDAI**	21.0 (± 9.3)	25.3 (± 12.6)	0.120
**CRP (mg/dL)**	10.7 (2.6‒21.9)	8.5 (3.9‒22.1)	0.956
**ESR (mm/hr)**	29.0 (9.5‒46.5)	22.0 (11.0‒34.8)	0.650

* Data are presented as mean (±standard deviation) or as number (%).

RTX, Rituximab; sDMARD, synthetic Disease-Modifying Antirheumatic Drugs; bDMARD, biologic Disease-Modifying Antirheumatic Drugs; DAS28, Disease Activity Score-28 for Rheumatoid Arthritis with Erythrocyte Sedimentation Rate; DAS28-CRP, Disease Activity Score-28 for Rheumatoid Arthritis with C-reactive protein; CDAI, Clinical Disease Activity Index; SDAI, Simple Disease Activity Index; CRP, C-Reactive Protein; ESR, Erythrocyte Sedimentation Rate.

Anti-CCP data were available for 34/40 (85%) in the RTX-GP2013 group and 49/67 (73%) in the RTX-originator group.

Regarding disease characteristics, a high frequency of seropositivity for rheumatoid factor or anti-CCP was observed in both groups, with no significant differences (p > 0.05). However, the RTX-GP2013 group presented nearly half of the frequency of extra-articular manifestations compared with the RTX-originator (20.0% vs. 43.3%, p = 0.020). In addition, the use of concomitant conventional DMARDs was similar in both groups (p > 0.05), but patients in the RTX-GP2013 group had a higher frequency of prior advanced therapies than the RTX-originator group (p < 0.001) (Table 1).

Parameters such as DAS28-ESR, DAS28-CRP, SDAI, ESR, and CRP were comparable between the two groups at baseline (p > 0.05) (Table 1). The only difference observed was in the CDAI score, which was lower among patients receiving the RTX-GP2013 in comparison to RTX-originator (19.1 [±9.5] vs. 23.6 [± 12.1], p = 0.047).

### Disease activity parameters at six months

The assessment conducted after the first RTX cycle also demonstrated comparable disease activity indices between groups, including CDAI (p > 0.05) (Table 2). The proportion of patients achieving remission/low disease activity was also similar between the groups, based on DAS28-CRP, SDAI, and CDAI criteria (p > 0.05).

**Table 2:** Disease activity parameters of RA patients at 6-months.

	RTX-GP2013	RTX-originator	
	n = 40	n = 67	p
**Prednisone dose (mg/day)**	5.0 (5.0‒10.0)	5.0 (5.0‒10.0)	0.715
**Number of patients who reduced prednisone dose**	20 (50.0%)	43 (64.2%)	0.161
**DAS28-ESR**	4.3 (± 1.4)	3.9 (± 1.6)	0.111
**DAS28-CRP**	4.0 (± 1.4)	3.7 (± 1.4)	0.277
**CDAI**	17.4 (± 11.8)	15.6 (± 14.2)	0.212
**SDAI**	19.1 (± 12.6)	17.0 (± 14.6)	0.176
**CRP (mg/dL)**	8.0 (3.4‒22.2)	7.0 (3.4‒13.9)	0.436
**ESR (mm/hr)**	21.0 (12.3‒33.3)	16.0 (10.0‒34.0)	0.375
**Remission/LDA (DAS28-CRP)**	10 (25.0%)	29 (43.2%)	0.064
**Remission/LDA (SDAI)**	10 (25.0%)	30 (44.8%)	0.062
**Remission/LDA (CDAI)**	12 (30.0%)	32 (47.8%)	0.103
**Infusion-related reactions**	7 (17.5%)	6 (9.0%)	0.231
**Drug retention (second RTX cycle)**	31 (77.5%)	57 (85.1%)	0.433

*Data are presented as mean (±standard deviation) or as number (%).

RTX, Rituximab; DAS28, Disease Activity Score-28 for Rheumatoid Arthritis with Erythrocyte Sedimentation Rate; DAS28-CRP, Disease Activity Score-28 for Rheumatoid Arthritis with C-reactive protein; CDAI, Clinical Disease Activity Index; SDAI, Simple Disease Activity Index; CRP, C-Reactive Protein; ESR, Erythrocyte Sedimentation Rate; LDA, Low Disease Activity [DAS28-CRP (< 3.2), CDAI (≤ 10), and SDAI (≤ 11)].

The univariate and multivariable analysis (Table 3) identified a lower number of prior advanced therapies (p = 0.003), as well as lower baseline scores of DAS28-CRP (p = 0.003), CDAI (p = 0.003), SDAI (p = 0.002), and CRP levels (p = 0.031), as independent predictors of remission/low disease activity at 6 months based on CDAI. In contrast, disease duration, extra-articular manifestations, and prior or concomitant sDMARDs were not significantly associated with response. Importantly, the type of RTX (RTX-GP2013 or RTX-originator) was not independently associated with clinical response at 6 months (p = 0.265), and effect estimates were consistent with clinical non-inferiority of the biosimilar compared with the originator. Because disease activity indices (DAS28, DAS28-CRP, CDAI, SDAI, CRP, and ESR) are strongly correlated, separate models were explored in the multivariable analysis to avoid multicollinearity.

**Table 3:** Baseline predictors of low disease activity according to CDAI.

	CDAI ≤ 10	CDAI > 10		Multivariate analysis	
	n = 40	n = 67	p	Odds Ratio	p
**RTX-GP2013 use (vs. originator)**	30 (80.0%)	37 (55.0%)	0.062	1.85 (0.63‒5.45)	0.265
**Age (years)**	57.3 (± 11.6)	55.0 (± 11.8)	0.322		
**Disease duration (years)**	14.7 (± 9.6)	17.0 (± 9.1)	0.219		
**Rheumatoid Factor +**	37 (92.5%)	65 (97.0%)	0.360		
**AntiCCP +**	27/33 (81.8%)	45/50 (90.0%)	0.332		
**Extra-articular manifestation**	19 (47.5%)	20 (29.9%)	0.097	1.39 (0.53‒3.65)	0.504
**Number of previous sDMARDs**	3 (2‒4)	3 (2‒4)	0.782		
**Number of previous bDMARDs**	1 (0‒2)	2 (1‒4)	<0.001	0.56 (0.38‒0.82)	**0.003**
**Concomitant use of:**					
Methotrexate	14/40 (35.0%)	26/67 (38.8%)	0.837		
Leflunomide	13/40 (32.5%)	19/67 (28.4%)	0.668		
Prednisone dose	10 (5‒15)	7.5 (5‒10)	0.126		
**DAS28**	4.1 (± 1.4)	5.1 (± 1.3)	<0.001	0.18 (0.03‒1.14)	0.069
**DAS28-CRP**	3.9 (± 1.1)	4.7 (± 1.1)	<0.001	0.49 (0.30‒0.79)	**0.003**
**CDAI**	17.4 (± 9.6)	24.5 (± 11.6)	0.001	0.93 (0.88‒0.97)	**0.003**
**SDAI**	18.7 (± 9.8)	26.7 (± 11.6)	<0.001	0.92 (0.88‒0.97)	**0.002**
**CRP (mg/dL)**	7.2 (3‒11.4)	12.6 (3.0‒26.1)	0.020	0.97 (0.95‒0.99)	**0.031**
**ESR (mm/hr)**	17.0 (8‒28.5)	26.0 (12.5‒44.5)	0.016	0.99 (0.97‒1.02)	0.602

* Data are presented as mean (±standard deviation) or as number (%).

RTX, Rituximab; sDMARD, synthetic Disease-Modifying Antirheumatic Drugs; bDMARD, biologic Disease-Modifying Antirheumatic Drugs; DAS28, Disease Activity Score-28 for Rheumatoid Arthritis with Erythrocyte Sedimentation Rate; DAS28-CRP, Disease Activity Score-28 for Rheumatoid Arthritis with C-reactive protein; CDAI, Clinical Disease Activity Index; SDAI, Simple Disease Activity Index; CRP, C-Reactive Protein; ESR, Erythrocyte Sedimentation Rate.

The 6-month retention rate, defined as continuation of treatment when a subsequent cycle was indicated, was 77.5% in the RTX-GP2013 group and 85.1% in the RTX-originator group, with no significant difference (p = 0.433), as shown in Table 2.

Regarding the infusion reactions, no significant difference was observed between the two groups, with rates of 17.5% in the RTX-GP2013 group and 9.0% in the RTX-originator group (p = 0.231) (Table 2). All reactions were classified as mild or moderate.

## Discussion

This study provides new real-world evidence for the comparability of efficacy and safety between the RTX-GP2013 and RTX-originator, specifically evaluating the response to the first treatment cycle in RTX-naïve patients with RA.

Among the strengths of the study are its real-world design, conducted at a single tertiary referral center, which ensured consistency in clinical management. All patients had treatment indications based on joint involvement, and a standardized institutional protocol with predefined timepoints and validated metrics of disease activity that ensured consistent and reliable data collection. Another strength is the focus on RTX-naïve patients, a group less frequently represented in real-world biosimilar research.

This study has some limitations that should be considered when interpreting the results. The sample size, inherent to a single-center real-world study, may have limited the ability to detect statistically significant differences between groups, particularly for outcomes with moderate effect sizes. Even several years before the replacement of RTX-originator with the biosimilar by the public healthcare system, the medication had already faced recurrent periods of shortage, which compromised treatment continuity and the regular administration of six-month cycles. Due to this, the authors chose to evaluate only the first treatment cycle of each medication to avoid interference from factors such as delays between doses. However, this approach restricted the evaluation of long-term outcomes, a wider range of adverse events, and immunogenicity, including the assessment of anti-drug antibodies.

Nevertheless, another limitation was the distinct period for inclusion of each drug. This temporal difference reflects national procurement policies, in which the biosimilar replaced the originator in the public healthcare system. As a result, patients in the RTX-GP2013 group were older and more treatment-refractory, likely reflecting the greater availability of alternative therapies in more recent years and the influence of local treatment guidelines,^40^ which recommend RTX primarily for refractory cases. Accordingly, a numerical trend toward higher remission/LDA rates was observed in the RTX-originator group across disease activity indices; however, these differences did not reach statistical significance and likely reflect baseline imbalances in disease severity and prior treatment exposure.

In this context, despite these baseline differences, the present results demonstrated comparable clinical response and safety between the two agents. Multivariate analysis was performed to mitigate baseline imbalances, although residual confounding cannot be fully excluded. Therefore, although this study was not designed as a randomized non-inferiority trial, the absence of clinically meaningful differences, together with overlapping confidence intervals, supports the interpretation of clinical comparability between RTX-GP2013 and the originator in RTX-naïve RA patients treated in routine practice.

Randomized controlled trials^9^, ^10^, ^11^, ^12^, ^13^, ^14^, ^15^, ^16^, ^17^, ^18^, ^19^, ^20^, ^21^, ^22^, ^23^, ^24^, ^25^, ^26^, ^27^ have previously confirmed the equivalent efficacy of RTX biosimilars and the originator. However, real-world evidence remains limited,^32^, ^33^, ^34^, ^35^, ^36^, ^37^, ^38^ and most reports focus on patients switched from the originator to biosimilar. These switched studies reported reassuring one-year drug retention rates ranging from 81.5% to 88%^31^^,^^33^^,^^36^^,^^37^ but without comparison to the originator.^31^^,^^36^ This study addresses this gap by directly comparing the two formulations in the same cohort and focusing on the first treatment cycle. The authors observed similar indications for retreatment, underscoring the equivalence of both formulations in clinical practice. Discontinuation in both groups was mainly driven by primary treatment failure, occurring in 67% of patients in the RTX-biosimilar group and 70% in the RTX-originator group.

Another relevant observation is the apparent discrepancy between response rates and treatment retention. Although only about one-third of patients in the biosimilar group achieved low disease activity, more than 75% continued to a second cycle. This suggests that treatment decisions were not driven solely by formal response indices but also by broader clinical judgment, physician and patient confidence, and limited therapeutic alternatives, factors that are highly relevant in real-world practice.

The safety results were fully consistent with previous reports, showing no significant difference in infusion-related reactions between RTX-GP2013 and RTX-originator groups. All reactions were mild or moderate. This aligns with data from Bahap-Kara et al.^34^ who reported similar safety profiles for CT-P10 and the originator, and from Smolen et al.,^19^ who reported approximately 15% infusion reaction rates for both agents.

Beyond individual clinical outcomes, RTX biosimilars contribute to healthcare sustainability by reducing costs and expanding access to high-cost biological therapies, particularly in resource-constrained settings. Increased market competition further supports broader and more equitable access.^6^^,^^7^ Gulácsi et al. estimated that adoption of the RTX biosimilar (CT-P10) in the European Union could save over €90 million in the first year, enabling treatment for more than 7,500 additional patients. These data underscore the economic and public health benefits of biosimilars.^8^

In conclusion, this real-world study provides additional evidence supporting the comparability of effectiveness, safety, and treatment retention between the rituximab biosimilar GP2013 and the originator product in rituximab-naïve patients with RA. Despite baseline differences reflecting a more treatment-refractory population in the biosimilar group, no clinically meaningful differences in disease activity outcomes, retreatment indication, or infusion-related reactions were observed, within the limits of the present sample size. These findings are consistent with clinical equivalence between the RTX biosimilar and the originator and reinforce the role of RTX biosimilars as effective and safe therapeutic options in routine clinical practice, contributing to broader access to biologic therapies without compromising patient outcomes.

## Ethical approval

This study was approved by the Local Ethics Committee on Human Research of the University of São Paulo (CAPPesq), under number 1298/06. Informed consent to participate in the study was obtained from all participants.

## Data availability statement

The datasets used and/or analyzed during the current study are available from the corresponding author on reasonable request.

## Authors’ contributions

All authors contributed to the study conception and design. Material preparation, data collection and analysis were performed by Barbara Bayeh, Fanny Reyes-Neira, Ana Cristina de Medeiros-Ribeiro and Andrea Yukie Shimabuco. The first draft of the manuscript was written by Barbara Bayeh and Andrea Yukie Shimabuco, and all authors commented on previous versions of the manuscript. All authors read and approved the final manuscript.

## Funding

This work was supported by grants from Fundacao de Amparo a Pesquisa do Estado de São Paulo (FAPESP #2015/03756-4 to E.B.) and Conselho Nacional de Desenvolvimento Cientifico e Tecnologico (CNPq grants #305068/2014-8 to E.B.).

## Declaration of competing interest

The authors declare no conflicts of interest.
